# Extracapsular Dissection Versus Superficial and Total Parotidectomy for Benign Parotid Tumours: A Retrospective Single-Centre Study

**DOI:** 10.7759/cureus.94084

**Published:** 2025-10-07

**Authors:** Mohamed Elmarghani, Iain Nixon

**Affiliations:** 1 Otolaryngology-Head and Neck Surgery, St John's Hospital at Howden, Edinburgh, GBR; 2 Otolaryngology, University of Edinburgh, Edinburgh, GBR

**Keywords:** benign parotid tumors, clinical outcomes, extra-capsular dissection, facial nerve palsy, parotidectomy complications, total parotidectomy

## Abstract

Extracapsular dissection (ECD) has emerged as a less invasive alternative to conventional parotidectomy techniques for the management of benign parotid tumours. This retrospective, single-centre study in Scotland, conducted between January 2015 and December 2022, compared the clinical outcomes of ECD with those of superficial (SP) and total parotidectomy (TP) across 55 patients treated by a single surgeon. Thirty-four patients underwent ECD, 17 SP, and 4 TP. Data on pathology, complications, surgical margins, and recovery were analysed, with the Freeman-Halton extension of Fisher's exact test applied to compare ECD and SP outcomes (p < .01). Patients were followed up for a mean duration of 4.8 years (median 4.5 years, range 1-9.5 years). ECD demonstrated favourable results, with lower rates of temporary facial nerve palsy (15%) compared to SP (35%) and TP (75%), and only one case of persistent palsy, noted in the ECD group. Although seromas were more frequent following ECD (18%), no haematomas or recurrences were observed in any cohort. Same-day discharge was achieved in 82% of ECD cases, significantly higher than SP (6%) and TP (0%) (p < .00001). Capsule integrity and tumour-free margins exceeded 88% across all groups, with no significant intergroup differences. Despite a smaller average tumour size in the ECD group, this did not affect outcomes. Overall, ECD provided equivalent oncological safety with superior recovery metrics, supporting its selective use in benign parotid disease.

## Introduction

Benign tumours of the parotid gland, predominantly pleomorphic adenomas and Warthin’s tumours, account for the majority of salivary gland neoplasms. Surgical excision remains the principal mode of management, aiming to prevent tumour progression and mitigate the risk of malignant transformation. Historically, superficial parotidectomy (SP) and total parotidectomy (TP) have been the standard surgical techniques. These approaches typically involve formal identification and dissection of the facial nerve, a process that, while ensuring oncological safety, carries a recognised risk of postoperative complications, notably transient or permanent facial nerve palsy, Frey’s syndrome, and aesthetic deformity due to soft tissue disruption.

In response to these concerns, extracapsular dissection (ECD) has emerged as a minimally invasive alternative, particularly suited to small, mobile, and superficially located benign tumours. ECD entails tumour excision with a margin of surrounding normal tissue, without necessitating full exposure of the facial nerve. Proponents of ECD suggest that this technique may reduce operative time and surgical morbidity while preserving oncological efficacy. Multiple studies have reported favourable outcomes with ECD. Kato et al. observed that ECD was associated with shorter operative duration, reduced inpatient stay, and lower healthcare costs compared with conventional parotidectomy techniques, without compromising tumour control [1]. Furthermore, Foresta et al. found that ECD was linked to a significantly lower incidence of facial nerve dysfunction and Frey’s syndrome than SP, supporting its use in selected cases [2]. Additional support for the safety and effectiveness of ECD comes from institutions such as the Vienna Medical School, where Kadletz et al. demonstrated comparable oncological outcomes to SP, with decreased morbidity [3]. Similarly, McGurk et al. reported that ECD achieved tumour control with reduced facial nerve morbidity and improved recovery times [4]. A meta-analysis by Albergotti et al. further validated these findings, highlighting the reduced complication rates associated with ECD across multiple studies [5].

Despite these advantages, questions remain regarding the adequacy of oncological margins and the long-term recurrence rates associated with ECD, particularly when lesions are in close proximity to the facial nerve. Moreover, same-day discharge following parotid surgery, an increasingly valued feature of modern surgical practice due to its cost-effectiveness and patient satisfaction, appears to be more feasible following ECD than after more extensive procedures. Emerging evidence suggests that minimally invasive approaches such as ECD may facilitate earlier discharge without compromising patient safety [1].

In light of these considerations, the present study aims to compare the clinical outcomes of ECD, SP, and TP in the surgical management of benign parotid tumours. The primary objectives are to evaluate the incidence of facial nerve palsy and the feasibility of same-day discharge. Secondary objectives include assessment of complication rates, margin status, and early recurrence. By analysing these outcomes within a single-centre, single-surgeon cohort, this study seeks to contribute to the growing body of literature on the safety and efficacy of less invasive parotid surgery.

## Materials and methods

A retrospective review was undertaken of patients undergoing surgery for benign parotid disease at a single tertiary otolaryngology centre in Scotland between January 2015 and December 2022. The study cohort comprised 55 patients, each of whom underwent one of three surgical procedures: extracapsular dissection (ECD), superficial parotidectomy (SP), or total parotidectomy (TP). Specifically, 34 patients underwent ECD, 17 underwent SP, and four underwent TP. All procedures were performed by a single consultant head and neck surgeon to ensure consistency in surgical technique and case selection. In our single-centre series, the mean follow-up duration was 4.8 years (median 4.5 years, range 1-9.5 years). Inclusion criteria encompassed adult patients (≥18 years) with histologically confirmed benign parotid neoplasms who had complete operative, pathological, and follow-up records. Exclusion criteria were malignant pathology, previous parotid surgery, and incomplete clinical data.

Patient records were reviewed using the hospital’s electronic health record system. Data collected comprised patient demographics (age and sex), histopathological diagnosis, tumour size as determined by histopathological examination, surgical technique, intraoperative findings, capsule integrity, and margin status. Postoperative variables included complications (e.g., facial nerve palsy, haematoma, seroma), duration of hospital stay, and recurrence during follow-up. Facial nerve function was assessed immediately postoperatively and at follow-up appointments. Palsy was classified as either transient (resolved within six months) or persistent (lasting beyond six months), based on clinical examination findings documented in follow-up notes.

The primary outcomes of interest were the incidence of facial nerve palsy and the rate of same-day discharge. Secondary outcomes included postoperative complications, early recurrence rates, and oncological margin status. Given the small number of TP cases, statistical analysis was primarily focused on comparing the ECD and SP groups. Statistical analyses were performed using Microsoft Excel and R software, version 4.2.2 (R Foundation for Statistical Computing, Vienna, Austria). Categorical variables were compared using the Chi-square test or Fisher’s Exact test, with the Freeman-Halton extension applied for small sample sizes. Continuous variables, including tumour size, were assessed using F-tests and analysis of variance (ANOVA). A two-sided p-value of <0.05 was considered statistically significant. All data were anonymised prior to analysis, and the study was conducted in accordance with institutional clinical governance guidelines. As a retrospective review of anonymised data, formal ethical approval was not required under UK Health Research Authority regulations.

## Results

A total of 55 cases were analysed with the following distribution of surgical procedures: Extracapsular Dissection (ECD) accounted for 34 cases (62%), Superficial Parotidectomy (SP) for 17 cases (31%), and Total Parotidectomy (TP) for four cases (7%). Table 1 shows patient demographics, pathology, complications, and surgical outcomes by type of parotidectomy.

**Table 1:** Patient demographics, pathology, complications, and surgical outcomes by type of parotidectomy Statistical significance was assessed using Fisher’s Exact test for categorical variables where expected counts were small. Chi-square (χ²) values are reported in the “Statistical significance” column where applicable. SPA: Sclerosis polycystic adenosis.

Variable	Extracapsular Dissection (n=34, 62%)	Superficial Parotidectomy (n=17, 31%)	Total Parotidectomy (n=4, 7%)	Statistical significance
Pathology				
Pleomorphic adenoma	30 (88%)	12 (71%)	3 (75%)	>0.05
Cysts (all types)	3 (9%)	1 (6%)	0	NA
Warthin’s tumour	0	4 (24%)	1 (25%)	NA
Sclerosing polycystic adenosis	1 (3%)	0	0	NA
Complications				
Facial nerve (FN) palsy (%)	6 (16%)	6 (35%)	3 (75%)	<0.05 (χ²=2.65)
└ Persistent FN palsy	1	0	0	NA
└ Temporary FN palsy	5	6	3	NA
Hematoma	0	0	0	NA
Seroma	6 (18%)	1 (6%)	0	>0.05 (χ²=1.32)
Recurrence	0	0	0	NA
Same-day discharge	28 (82%)	1 (6%)	0	<0.05 (χ²=27.02)
Demographics				
Female	20 (59%)	12 (70%)	2 (50%)	>0.05
Male	14 (41%)	5 (30%)	2 (50%)	>0.05
Surgical margins				
Capsule integrity	32 (94%)	14 (82%)	4 (100%)	>0.05 (χ²=1.77)
Tumour at margin (cyst & SPA excluded)	28 (93%)	14 (88%)	4 (100%)	>0.05 (χ²=0.45)

Pathological findings

Pleomorphic adenoma was the most frequent diagnosis, observed in 88% of ECD cases, 71% of SP cases, and 75% of TP cases. Cysts of various types occurred in 9% of ECD and 6% of SP but were absent in TP. Warthin’s tumour occurred exclusively in SP (24%) and TP (25%), while one case of sclerosing polycystic adenosis was identified in the ECD group. Differences in pathology were not statistically significant (p = 0.264).

Facial nerve outcomes

Facial nerve (FN) palsy occurred in 16% of ECD cases, 35% of SP cases, and 75% of TP cases. This difference was statistically significant (p = 0.0366, Fisher’s Exact Test). Persistent facial nerve palsy was observed in one patient in the ECD group, with no cases reported in the SP or TP groups. The palsy was classified as House-Brackmann grade 3 and remained unchanged throughout the follow-up period. Temporary FN palsy was more common following SP and TP, though this was not independently tested for significance. The use of Fisher’s Exact Test rather than Chi-square was considered more reliable given the small subgroup sizes.

Postoperative complications

No cases of haematoma were reported in any group. Seroma occurred in 18% of ECD cases, in one SP case (6%), and in none of the TP cases. This difference was not statistically significant (p = 0.533). No recurrences were observed during follow-up.

Same-day discharge

Same-day discharge was achieved in 82% of ECD patients, but in only 6% of SP patients and none of the TP patients. This difference was highly statistically significant (p < 0.00001), indicating a strong association between surgical approach and discharge feasibility.

Demographics

Female patients comprised 59% of ECD cases, 70% of SP cases, and 50% of TP cases. The distribution of gender did not differ significantly between groups (p = 0.610).

Surgical margins and capsule integrity

Capsule integrity was preserved in 94% of ECD, 82% of SP, and 100% of TP cases. This difference was not statistically significant (p = 0.422). When cysts and sclerosing polycystic adenosis were excluded, tumour-free margins were achieved in 93% of ECD, 88% of SP, and 100% of TP cases (p = 1.000) (Table 2). No recurrences were identified during the study period.

**Table 2:** Average tumour measurements by surgical approach with corresponding statistical significance

Variable	Extracapsular Dissection (n: 27)	Superficial Parotidectomy (n: 10)	Total Parotidectomy (n: 2)	P value	F Value (ANOVA)
Average tumour measurement at peak	Mean 19.4 SD: 6.63	Mean 22.08 SD: 11.59	Mean 22.45 SD: 11.43	0.0079	5.55

Tumour size

After excluding cysts, sclerosing polycystic adenosis, and cases without recorded measurements, the mean tumour diameter was 19.4 mm (SD 6.63) for ECD, 22.1 mm (SD 11.6) for SP, and 22.5 mm (SD 11.4) for TP. This difference was statistically significant (p = 0.0079, ANOVA F = 5.55), confirming that smaller tumours were preferentially managed with ECD. This is demonstrated in Table 2 above.

## Discussion

This single-centre series supports the growing consensus that ECD provides significant benefits in the management of benign parotid tumours. Our results show that ECD is associated with superior preservation of facial nerve function and a markedly greater likelihood of same-day discharge compared with SP and TP, while oncological outcomes remain comparable. These findings are consistent with the broader contemporary literature indicating that carefully selected, well-circumscribed lesions can be managed with gland-preserving approaches without compromising disease control. A recent single-team comparative analysis likewise reported significantly fewer overall complications with ECD than SP (Fisher’s Exact, p < 0.05), supporting the nerve-sparing profile of limited dissection in eligible patients [6].

Facial nerve palsy is among the most impactful complications following parotid surgery. The reduced rates observed in our ECD cohort align with previous findings demonstrating that limited dissection minimises nerve traction and thermal injury [2-4]. A machine-learning-assisted synthesis summarised transient FN palsy rates across techniques and again highlighted operative extent, tumour factors, and prior surgery as key drivers of risks, findings that support a strategy of minimising dissection when oncologically safe [7]. Large prospective series have similarly reported transient palsy in around 15% and permanent palsy in fewer than 5% of benign parotid resections, with ECD associated with the lowest risks [8,9]. More recent multicentre studies have reinforced the correlation between surgical extent and postoperative nerve weakness, highlighting the safety of less invasive techniques when applied in selected cases [9]. The lower rates of facial nerve palsy observed with ECD were supported by Fisher’s Exact Test, suggesting that these differences may not be solely due to small-sample variation.

Same-day discharge following parotidectomy is increasingly advocated as part of enhanced recovery and ambulatory pathways. Our study found that over 80% of ECD patients were discharged the same day, compared with only one SP case and none of the TP cohort. This mirrors the findings of Kato et al. [1] and Flach et al. [10], who reported high outpatient feasibility for ECD. A systematic review and meta-analysis likewise concluded that outpatient parotidectomy is safe, with complication and readmission rates equivalent to inpatient surgery [10]. International prospective studies have also demonstrated that the extent of dissection is closely tied to the risk of nerve palsy and other late complications such as Frey’s syndrome, strengthening the case for minimally invasive approaches in well-selected cases [9,11]. In addition, multicentre outcome studies have shown that ambulatory management can improve cost-efficiency without compromising safety [12,13].

Concerns regarding oncological safety remain important; however, our results demonstrated high capsule integrity and margin clearance rates across all surgical approaches, with no early recurrences observed during follow-up. These findings accord with long-term reports demonstrating low recurrence after ECD in well-selected patients [8,9]. The significantly smaller tumour sizes in the ECD cohort reflect cautious case selection rather than technical limitation. Careful preoperative imaging and intraoperative assessment remain essential to ensure appropriate patient selection.

Several limitations must be acknowledged. The retrospective design, single-surgeon series, and small sample size introduce case selection and reporting biases, as well as subgroup comparisons, particularly those involving TP, which lack statistical power. Follow-up was of limited duration, meaning that long-term recurrences could not be fully assessed. Additionally, as ECD is a technique that requires surgical expertise, our favourable outcomes may not be generalisable to low-volume centres. Nevertheless, the strengths of this study include uniform surgical technique, consistent follow-up, and the application of statistical methods appropriate for small samples, which together lend credibility to the observed associations.

In summary, and recognising the limitations of this study, our findings contribute to the growing body of evidence suggesting that ECD may be a safe, effective, and resource-efficient alternative to conventional parotidectomy. Its potential advantages in preserving facial nerve function and facilitating same-day discharge indicate that it could be a valuable option in modern parotid surgery, particularly when careful case selection and appropriate surgical expertise are applied.

## Conclusions

Extracapsular dissection may represent a safe and effective alternative to conventional parotidectomy for benign, well-circumscribed parotid tumours. It appears to offer favourable facial nerve preservation and the possibility of same-day discharge, with oncological outcomes that are broadly comparable to more extensive procedures. While the limitations of sample size and single-centre design must be acknowledged, these findings suggest that ECD could be considered in carefully selected patients. Prospective multicentre studies are needed to further refine selection criteria and confirm long-term outcomes.
